# The Family Check-Up 4 Health (FCU4Health): Applying Implementation Science Frameworks to the Process of Adapting an Evidence-Based Parenting Program for Prevention of Pediatric Obesity and Excess Weight Gain in Primary Care

**DOI:** 10.3389/fpubh.2018.00293

**Published:** 2018-10-15

**Authors:** Justin D. Smith, Cady Berkel, Jenna Rudo-Stern, Zorash Montaño, Sara M. St. George, Guillermo Prado, Anne M. Mauricio, Amanda Chiapa, Meg M. Bruening, Thomas J. Dishion

**Affiliations:** ^1^Department of Psychiatry and Behavioral Sciences, Northwestern University Feinberg School of Medicine, Chicago, IL, United States; ^2^Department of Preventive Medicine, Northwestern University Feinberg School of Medicine, Chicago, IL, United States; ^3^Department of Pediatrics, Northwestern University Feinberg School of Medicine, Chicago, IL, United States; ^4^REACH Institute, Department of Psychology, Arizona State University, Tempe, AZ, United States; ^5^Phoenix Children's Hospital, Phoenix, AZ, United States; ^6^Children's Hospital of Los Angeles, University of Southern California, Los Angeles, CA, United States; ^7^Department of Public Health Sciences, University of Miami Miller School of Medicine, Miami, FL, United States; ^8^Yale Child Study Center, New Haven, CT, United States; ^9^Department of Nutrition, Arizona State University, Tempe, AZ, United States; ^10^Oregon Research Institute, Eugene, OR, United States

**Keywords:** adaptation, implementation strategies, family check-up, family check-up 4 health, obesity prevention, primary care, scaling out

## Abstract

Implementation experts have recently argued for a process of “scaling out” evidence-based interventions, programs, and practices (EBPs) to improve reach to new populations and new service delivery systems. A process of planned adaptation is typically required to integrate EBPs into new service delivery systems and address the needs of targeted populations while simultaneously maintaining fidelity to core components. This process-oriented paper describes the application of an implementation science framework and coding system to the adaptation of the Family Check-Up (FCU), for a new clinical target and service delivery system—prevention of obesity and excess weight game in primary care. The original FCU has demonstrated both short- and long-term effects on obesity with underserved families across a wide age range. The advantage of adapting such a program is the existing empirical evidence that the intervention improves the primary mediator of effects on the new target outcome. We offer a guide for determining the levels of evidence to undertake the adaptation of an existing EBP for a new clinical target. In this paper, adaptation included shifting the frame of the intervention from one of risk reduction to health promotion; adding health-specific assessments in the areas of nutrition, physical activity, sleep, and media parenting behaviors; family interaction tasks related to goals for health and health behaviors; and coordinating with community resources for physical health. We discuss the multi-year process of adaptation that began by engaging the FCU developer, community stakeholders, and families, which was then followed by a pilot feasibility study, and continues in an ongoing randomized effectiveness-implementation hybrid trial. The adapted program is called the Family Check-Up 4 Health (FCU4Health). We apply a comprehensive coding system for the adaptation of EBPs to our process and also provide a side-by-side comparison of behavior change techniques for obesity prevention and management used in the original FCU and in the FCU4Health. These provide a rigorous means of classification as well as a common language that can be used when adapting other EBPs for context, content, population, or clinical target. Limitations of such an approach to adaptation and future directions of this work are discussed.

## Introduction

Translation of evidence-based interventions, programs, and practices (EBPs) for children and adolescents to the real-world service systems that can support them is a challenging endeavor and the lack of wide scale dissemination and implementation is well documented (1, 2). EBPs grounded in the principles of parent training are highly effective at preventing a host of common mental and behavioral problems in youth (3) and have been found to be effective when tested under more “real-world” conditions (4). That is, conditions more closely aligned to typical operations and resources available in non-research settings. Parenting programs are slowly making their way into the service delivery systems where youth and families are served. These include social services, schools, and home visitation. A relevant setting where such interventions have not largely been adopted is pediatric primary care. This setting is particularly relevant for preventive parenting interventions as the majority of children in the U.S. receive annual primary care services (5); low-income children have high rates of access (6); parents expect to receive parenting advice from physicians and view them as respected experts; there are potentially stable mechanisms to fund these EBPs, whereas in other settings, these are lacking; and this setting does not hold the stigma that others, such as schools, do (7). Parenting in general, and the effects of parenting interventions specifically, are linked to both mental and physical health conditions, making these programs highly relevant as a primary prevention strategy for improving the health of all children (8). The existing primary care system is the ideal context for parenting interventions to be implemented. One of the barriers to doing so, however, is the need to adapt parenting programs for the primary care context and the populations that would receive these interventions.

Use of adaptation as an implementation strategy is common and aimed at making EBPs more appropriate, feasible, cost-effective, and acceptable for both the target population and service delivery system (9). Articles describing adaptations for particular populations are more common in the literature than are those for delivery through a new delivery system. The most common population adaptations are for different cultural groups. Appreciation for these adaptations grew as problems with focusing behavioral interventions exclusively on the majority culture, typically non-Latino White families, emerged. Specifically, focusing only on the majority culture often led to the EBP being ineffective with, or simply unpalatable to, culturally diverse populations (10–12). Adaptation to a new delivery system involves pursuing an alternative means through which to reach the target population. This form of adaptation has traditionally been done by changing the context through which an intervention is delivered (e.g., from schools to mental health or social service systems).

A traditional assumption in the field is that when EBPs are adapted, they need to be rigorously re-tested to ensure positive effects of the original program are not degraded. However, Chambers et al. (13) and others have argued that adaptation of EBPs—done in a way that maintains fidelity to the core components—should result in at least comparable effect sizes and are, perhaps more importantly, likely to be sustained. The results of a recent review of adapted EBPs by Wiltsey-Stirmen et al. (14) found little evidence that adaptations were detrimental to effectiveness. Relatedly, however, they also found limited consistent evidence that adapted protocols outperformed the originals; the exception was the addition of components, which had a modest positive impact on outcomes.

A process of “scaling out” has been recommended to more rapidly increase the reach of EBPs (15). *Scaling out* differs from the more common practice of *scaling up* an EBP, which means to spread to additional units of the same or a very similar context, and customarily targeting the same population, for which the EBP was originally tested and shown to be effective. When scale up occurs, there is an assumption that the EBP will be delivered in the same way to the same type of population or people and, therefore, health benefits will align with previous research if there is sufficient fidelity (16). It is thought that adaptation of the EBP either is unnecessary or will simply not occur in a meaningful way in a scale up scenario. In contrast, scaling out is defined by Aarons et al. (15) as a deliberate effort to adapt an EBP and broaden its delivery (a) to a different delivery system, but with the same target population as previous trials; (b) to a different target population, but within the same delivery system as previous trials; or (c) to a different target population and through a different delivery system than those of previous trials.

There are a number of process frameworks to prospectively guide the adaptation of EBPs, some of which are more generic (17, 18), while others are specific to cultural adaptation [see Barrera et al. (10)] or to technology-based platforms (19). Adapting an EBP for a new clinical target outcome goes beyond these models in important ways and is least represented in the literature. Aarons et al. (15) suggest that the “new target population” refers to the characteristics of the population, such as developmental period (i.e., age), culture, or socioeconomic status, but that the clinical target outcome is typically the same (e.g., a preventive intervention targeting problem behaviors in young children vs. adolescents). There are instances, however, when adapting an EBP for a new clinical target outcome may be warranted. The evidence for doing so may come from a number of potential sources, including studies examining collateral benefits of an intervention (i.e., effects on outcomes not directly targeted). For example, the Familias Unidas program was originally designed to prevent and reduce behavior problems and substance use in Latino adolescents, but it has also had positive effects on adolescents' internalizing symptoms and suicidal behaviors (20, 21). Another common collateral effect of parenting programs is improvement in parental mental health, such as reducing parents' depressive symptoms [e.g., Beach et al. (22), Shaw et al. (23)].

This article uses concepts and frameworks from the field of implementation research to present and document the process of scaling out an evidence-based parent training program for a new clinical target and service delivery system. The Family Check-Up [FCU; Dishion et al. (24)], which was originally tested in public schools, community mental health clinics, and home visiting services for families with youth at risk for problem behaviors, has been adapted to target the prevention of obesity and excess weight gain in collaboration with the primary healthcare system. This paper attempts to accomplish three aims: First, we propose four levels of evidence (minimum, preferred, preferred plus, and optimal) as a framework to guide decision-making around the adaptation of an EBP for a new clinical target—this is not represented in the adaptation literature. These levels pertain to the justification for conducting this type of adaptation. Second, we categorize the modifications and adaptations made to the FCU based on an existing framework, which was selected because it was developed in the context of implementation science, is comprehensive, and can be applied retrospectively (25). We describe our process by detailing the various methods and activities that were used to obtain salient guidance. These included analyses of existing data and reviews of the literature by the academic team, research-practice partnerships with local agencies, and collaboration with diverse community stakeholders. Activities with stakeholders comprised formal and informal meetings, a pilot study at a partner agency, establishing and regularly convening a community advisory board (CAB), and conducting a multisite randomized trial (currently ongoing) called the *Raising Healthy Children* study^1^ (26). Last, we apply a recent standardized taxonomy for specifying the behavior change techniques used in behavioral interventions for pediatric obesity (27) to the resulting adapted and enhanced version of the FCU, which we call the Family Check-Up 4 Health (FCU4Health), and contrast that with the original program components. This step is important in demonstrating that FCU4Health aligns with the characteristics of other EPBs for the prevention of pediatric obesity and excess weight gain. We anticipated that the process of adapting FCU for obesity prevention in primary care would center around changes and modifications to the content of the intervention to more specifically target weight-related variables and modifications to the delivery of the program to better align with the context of primary care, specifically aligning with the national recommendations for the prevention of excess weight gain and a staffing model consistent with the primary healthcare system.

### Proposed levels of evidence for adapting an EBI for a new clinical target

**Figure 1:**
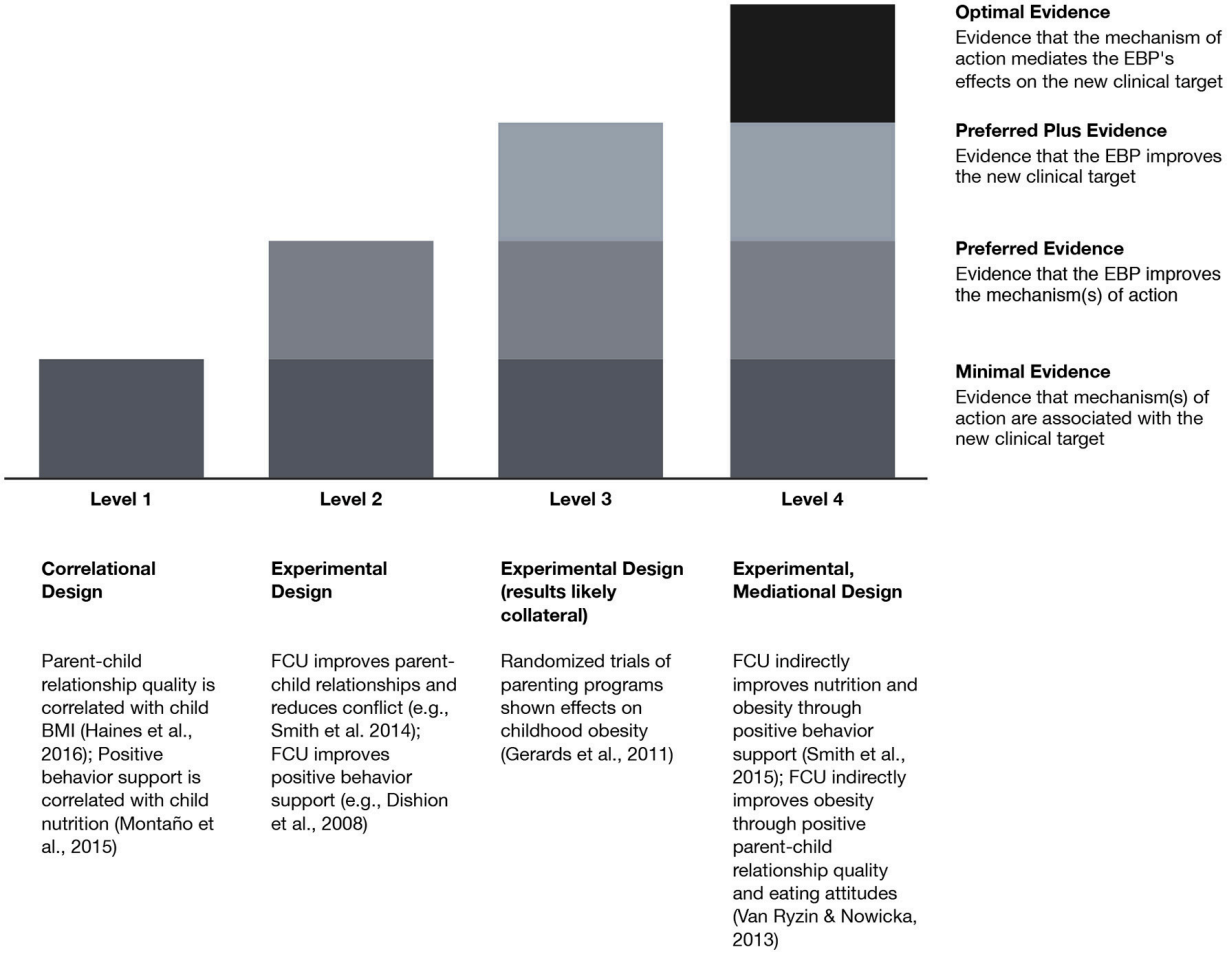
Levels of evidence for adapting evidence-based programs for a new clinical target: FCU for prevention of obesity and excess weight gain as an example. BMI, body mass index; EBP, evidence-based program; FCU, Family Check-Up.

Three of the authors (Smith, St. George, Prado) developed the proposed four levels of evidence to consider when endeavoring to adapt an EBP for a new clinical target (see Figure 1). The need to develop these levels of evidence emerged as these authors considered making adaptations for a new clinical target, which differs from adaptations for a new population or setting. With the large body of evidence indicating collateral effects of parenting interventions [see Van Ryzin et al. (28)], such a guide for adapters of these programs specifically for new clinical targets would be useful. Each level is cumulative; it requires the newly specified set of evidence in addition to the evidence listed in each of the previous levels. Although not necessary, it would be preferable each level of evidence be documented within the target population (e.g., Latino immigrants). If research with a specific target population is not available, this should not necessarily limit the adaptation of the EBP for the new clinical target. In situations where evidence in the target population is unavailable, researchers may want to consider whether (a) theory or input from relevant stakeholders *and* (b) cross-sectional OR (preferably) longitudinal research support the causal relations between the program, mechanisms of action, and the new clinical target. Experimental designs, such as randomized trials, are preferred at each level. Other designs (e.g., pre-post) are acceptable but multiple studies with consistent significant relations would be needed. In our descriptions of each level, we integrate information from our work with FCU as illustration. However, evidence can be garnered from different EBPs that have similar intervention strategies and theories of action (see Level 3 for an example of drawing from other EBPs to support adaptation of FCU).

#### Level 1: minimum evidence

To consider adapting an EBP for a new clinical target, it is important first to determine whether there is sufficient evidence demonstrating that the mechanisms of action (e.g., family functioning) of the EBP are related to the new clinical target (e.g., physical activity, weight loss). Such evidence would require that the EBP's mechanism(s) of action have been shown to influence the clinical target in more than one cross-sectional study or at least one longitudinal study. For example, family functioning has been found to be related to childhood obesity and obesity-related outcomes in both cross sectional (29) and longitudinal research (29–31).

#### Level 2: preferred evidence

This level requires all the evidence listed in Level 1 and documented evidence that the EBP impacts the mechanism(s) of action. For example, if a parenting intervention targeting substance use is being considered for adaptation to obesity or obesity-related outcomes, that EBP should have documented evidence that it leads to improvement in the mechanism(s) of action. Mediational analyses of randomized trials have demonstrated that the FCU prevents and reduces youth substance use through improvements on the same family processes that have been linked to obesity and obesity-related outcomes in longitudinal studies [e.g., (24, 32–38)].

#### Level 3: preferred plus evidence

This level requires the criteria listed above and evidence that the EBP has an impact on the new clinical target. There are a few examples of effects of parenting programs on obesity. For example, Brotman et al. (39) found that a parenting intervention not focused on improving physical health significantly reduced body mass index (BMI) 5 years post-intervention. (40) provide a review of similar effects of parenting programs on obesity.

#### Level 4: optimal evidence

This level requires the previous criteria plus evidence that the mechanism of action mediates the effects of intervention on the new clinical target. The original version of FCU, which was not designed to target obesity and obesity-related outcomes, has had collateral effects on obesity in two randomized clinical trials. In early childhood, effects on weight gain trajectories were mediated by immediate improvements in observed positive behavior support skills, which were in turn related to serving children more nutritious meals between the ages of 2 and 5 (41). This relationship between positive behavior support and nutrition was explored more granularly and found to be strongly related in early childhood in this trial (42). In adolescence, FCU effects on parent-child relationship quality had a positive impact on eating attitudes in late adolescence, which mediated the effects of the program on obesity rates (43).

Each of these levels provides evidence to support adapting an EBP for a new clinical target. Such adaptation of the intervention to the new clinical target, although not necessary, would likely yield stronger effect sizes with the content specifically related to the new outcome. For clarity in terminology, we henceforth use the term *adaptation* in reference to changes in the way FCU is delivered in primary care and the term *enhancement* in reference to additions and changes to the program's content in order to maximize potential impact on health behaviors related to obesity and excess weight gain [see Smith et al. (44)].

### Adaptation of the family check-up for the prevention of obesity and excess weight gain

The components and content of the original FCU model are described in Table 1. Additional information is available in Dishion and Stormshak (45) and Smith (46). In brief, the FCU involves a 3-step process comprising an initial interview with the family, an ecological family assessment (multimethod, multirater), and a motivation-enhancing feedback session. During the feedback session, family strengths, and areas for potential intervention identified in the ecological assessment are discussed with the caregiver(s) and motivational interviewing is used to motivate families to make change and engage in additional intervention. The primary form of subsequent intervention is behavioral parent training and a variety of community-based support services for the child (e.g., individual mental health intervention) and the caregiver(s) (e.g., marital or substance abuse counseling).

**Table 1:** Classifications of CONTEXT modifications to the original FCU in developing the FCU4Health program.

	**What is modified?**		**By whom were modifications made?**
	**FCU**	**FCU4Health**	
Format	1-on-1 Health maintenance model: Annual feedback sessions with individually-tailored family support and referral	1-on-1 Intensive: 3 feedbacks in 6 months with individually-tailored family support and referral (Goals: USPSTF recommendation of 25–50 h)	N/A Researchers (required by funding agency)
Setting	Schools Home visitation Community mental health Social services	Pediatric primary care (i.e., behavioral health service, integrated care) Home visitation	Program developers Agency administration
Personnel	Master's and doctoral-level mental health providers (e.g., social workers, psychologists) Referral: school, mental health provider, parent	Master's level providers in mental health (e.g., social workers) and related health care and health promotion fields (e.g., public health, nutrition) Referral: pediatrician	Program developers Agency administration Program developers Researchers (required by funding agency)
Population	Families with youth at risk for problem behaviors (e.g., oppositional, substance use, high risk sex, school failure) Low-income, underserved Ages 2–17 years (across multiple trials)	Families with youth at risk for obesity and excess weight gain (e.g., poor diet, low physical activity, racial/ethnic/cultural groups with disproportionate risk) Low-income, underserved Ages 6–12 in *Raising Healthy Children*	Program developers N/A N/A

**Figure 2:**
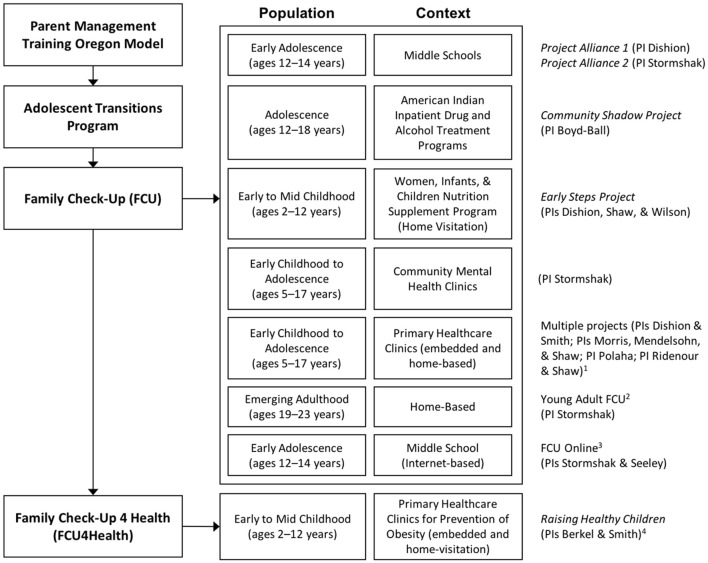
Characterizations and approximate chronology of adaptations to the FCU. ^1^Each project used an adaptation of the original FCU for the local context. ^2^This project is an extension of the *Project Alliance 2* cohort. ^3^FCU Online uses the content of original FCU. ^4^Berkel and Smith began a related project funded by the USDA in summer 2018.

Although adaptation of the FCU for the prevention of childhood obesity and excess weight gain and the primary care context has not been described previously, the program has been previously adapted for various populations and delivery systems. Figure 2 provides a schematic of the various adaptations of the FCU and the approximate chronology of these efforts. A critical facet of each adaptation is retention of the core components of the program and intervention strategies targeting age-appropriate parenting and behavior management skills [for further discussion, see Smith and Dishion (47). FCU was originally designed for the prevention of problem behaviors in the transition from middle to high school that increase the risk of substance use, high-risk sexual behaviors, violence, and other related outcomes (48) based on the successful Adolescent Transitions Program (49) and the Parent Management Training Oregon Model (50). The initial trials of the FCU occurred in public middle schools (children age 12 to 14 years) in underserved urban areas [see (35), (51)]. As described by Smith et al. (38), the FCU was designed to be multiculturally responsive and empirical studies have shown that different racial and ethnic groups participate in and benefit from the program similarly. However, there was not a specific adapted version of FCU for each racial/ethnic group; rather, the program was individually-tailored to the specific needs of each family [see Smith et al. (38)]. The first adaptation of FCU for a specific racial/ethnic group was for American Indian youth (52). Next, the program was adapted for families with young children ages 2 and 12 years (24) and for delivery through home visitation rather than embedded in schools (53). Then, FCU was adapted for delivery within community mental health clinics (54). More recently, the original FCU was adapted for delivery within and in coordination with primary healthcare systems (55). Ongoing studies are testing the effectiveness of (a) a version of the program adapted for emerging adults (ages 19 to 23 years) and their parents (56) and (b) an Internet-based delivery of FCU to families in rural areas identified in middle schools (57). In each of these situations, the context of FCU delivery and the population were targeted for adaptation, but the primary clinical target (i.e., reduction of child problem behaviors through the improvement of family management) remained consistent.

The developer of the FCU, Thomas Dishion, and other FCU researchers at multiple institutions have recently undertaken efforts to adapt the program to fit better within and in coordination with pediatric primary care. These efforts coincide with a national movement to implement evidence-based parenting programs within primary care for the prevention and the treatment of behavioral and mental health conditions (7, 8, 58). In addition to the effort described in this paper, Shaw et al. (59) have been working in partnership with primary care practices to reach children in need of family support services to prevent substance use in pre- and early adolescence and improve school readiness in young children. Their approach, however, involves less adaptation to the FCU itself as they identify eligible families in primary care, but then deliver the FCU through the previously successful home visitation model (outside of the primary care office). This linking of an EBP with the primary care context is important, but places fewer demands on the system to adopt the FCU compared to a more integrated delivery model, and therefore, there is lesser need to adapt the FCU for the demands of the context and is potentially more quickly translated. Evaluating this hypothesis is a primary aim of the ongoing *Raising Healthy Children* study (26) and a second study also being led by Smith and Berkel that is funded by the United Department of Agriculture (Grant number: 016–10799). Relatedly, Polaha and colleagues pilot tested the FCU in primary care clinics for young children's mental and behavioral concerns. The process and outcomes of these efforts are discussed in Smith et al. (60) and Smith and Polaha (55).

In addition to adapting the FCU for delivery in the primary care context, we also undertook a process of enhancing the program to better target health behaviors and parental supports to prevent obesity and excess weight gain. The findings of previous research, including our own with the original FCU, have resulted in efforts to enhance evidence-based parenting programs to more specifically address behaviors related to maintaining a healthy weight. Familias Unidas (St. George, S. M., Messiah, S. E., Sardinas, K. M., Poma, S., Lebron, C., Tapia, M.,…Prado, G. *Familias Unidas for health & wellness: Adapting an evidence-based substance use and sexual risk behavior intervention for obesity prevention in Hispanic adolescents*, submitted for publication) and Lifestyle Triple P (61) are other examples in the family intervention literature of adaptation for this new clinical target. The activities and sources of data used in the adaptation and enhancement of the FCU for the prevention of excess weight gain through primary care are presented in the Method section and how each contributed to the adapted and enhanced version of the program—the FCU4Health—is presented in the Results section.

## Methods

### Procedure

Adapting an EBP when scaling out should generally comprise an iterative, multi-method, and multi-informant process. Our process occurred through a variety of activities and sources of data. These are described in the chronological order in which they occurred. Continuation or repetition over time is noted as appropriate. This study was carried out in accordance with the United States Department of Health and Human Services (HHS) policy for the protection of human subjects. The protocols of the pilot study and ongoing *Raising Healthy Children* study from which data were drawn for this article were approved by the institutional review boards of Arizona State University and the Phoenix Children's Hospital. All subjects gave written informed consent in accordance with the Declaration of Helsinki.

#### Evidence from prior trials of FCU for adolescents

As summarized above and in Figure 1, there was optimal evidence for adapting the FCU to focus on health behaviors. Enhancement for the prevention of obesity and excess weight gain was informed by analyses of prior trials of the FCU showing mediated effects of the intervention on obesity and related processes (41, 43).

#### Meetings with pediatricians and social workers at phoenix children's hospital (PCH)

In 2011, FCU developer Dishion and collaborator Berkel began a series of formal and informal meetings with pediatricians and social work staff at PCH concerning the acceptability and appropriateness of using the FCU in general pediatrics. These meetings comprised presentations by Dishion and sharing of FCU materials. The chief of pediatrics and other clinic leadership also attended. This partnership was made possible by Berkel's dual appointment at ASU and PCH's general pediatrics care coordination program (Berkel, C., Araica, E., Smith, J. D., Tovar-Huffman, A., Beaumont, S. W., & Shaw, T. *Connecting families: Implementation and outcomes of a comprehensive care coordination program*, manuscript in preparation).

#### Pediatrician needs and attitudes survey

As a result of these meetings focused on exploring use of FCU in general pediatrics, in 2012, Berkel et al. (62) conducted a survey of the 20 physicians in the general pediatrics clinics about their concerns for families and attitudes toward implementing the FCU in the clinic. The top three areas of concern were *obesity* (100%), *nutrition* (95%), and *parenting* (90%). All respondents perceived a need for a program like the FCU that could also address family factors related to weight management. Open-ended responses, provided by 70% of respondents, reflected themes of limited time to convey important, tailored health information; the desire to increase parent understanding and empowerment to support children's health behavior change; and a recognition of the many barriers to families' being able to follow through with recommendations for healthy lifestyle behavior change. Further, pediatricians reported feeling unprepared to contend with these family-level barriers to follow through due to their lack of training in this area and their many practice demands. In 2011, Dishion and Berkel submitted a grant to test the effectiveness of the FCU when implemented by general pediatrics care coordinators in early childhood (birth to five). This grant was not funded. In 2014, Berkel, Dishion, and Smith submitted a grant to test the effectiveness of the FCU through care coordination with adolescents, and Smith, Marisol Perez, and Dishion submitted a grant to test FCU as an add-on parenting support service for families in an outpatient specialty care clinic for obesity in the hospital. Neither of these grants were funded.

#### Pilot trial of FCU in pediatric primary and specialty care

Dishion was awarded a seed grant from Arizona State University (ASU) to conduct a pilot feasibility trial of implementing the FCU in general pediatrics and a specialty clinic in the department of gastroenterology for children with non-alcoholic fatty liver disease at PCH. These two clinics were selected to obtain information about feasibility, appropriateness, and acceptability of FCU for outpatient primary and specialty care, and how the different population characteristics could inform adaptation of the program to better address prevention of obesity and excess weight gain across levels of disease progression. The pilot trial was run by Dishion, with assistance from Berkel, Smith, and ASU clinical psychology graduate students (Montaño, Rudo-Stern, Chiapa) who provided the FCU. Eleven families and fourteen families were consented and participated in some aspect of FCU in the fatty liver and general pediatrics clinics, respectively. The project demonstrated a need to adapt the FCU to fit clinic procedures, which necessitated adaptations to program delivery and addition of a relevant screening process and instrument (63). Activities followed a participatory research approach with health care staff (pediatricians, nurses, dieticians), patients, and their families. All eligible families were offered the FCU at no charge and received a $20 gift card for completing the assessments. Qualitative findings from stakeholder interviews with pediatricians (*n* = 11) and dieticians (*n* = 4) indicated that these stakeholders: (1) desired to involve families in the FCU; (2) actively offered FCU to families; (3) saw a need for an intervention to help families with parenting practices as they relate to children's physical health; and (4) felt the name “Family Check-Up” is appropriate in this setting. Additionally, the average score on the Evidence Based Practice Attitudes Scale (64) reported by physicians and dietitians (*n* = 15) was a 44 (out of 50) indicating adequate acceptability. Results of family interviews revealed (1) receptivity to the family-centered nature of the FCU to support parents; (2) parents see themselves as the primary source of support for their children; (3) acceptability of a program that supports parents in implementing treatment recommendations; (4) if an intervention that provided this type of additional support for parents was being offered in their clinic, they would find it appealing and would enroll; and (5) the name “Family Check-Up” is appropriate in this setting. Individual outcomes were not assessed as part of this trial as the goal was determining feasibility and needed adaptations and enhancements.

#### Raising healthy children study: hybrid effectiveness-implementation trial in primary care

In 2014, Smith, Perez, Dishion, and others submitted a proposal for federal funding to conduct a randomized trial of the FCU in specialty care for the management of obesity. The proposal was not funded. In 2015, Berkel and Smith led a proposal in response to a request for applications issued by the Centers for Disease Control and Prevention under the Childhood Obesity Research Demonstration (CORD) projects, version 2.0 announcement. The proposal was selected for funding and the *Raising Healthy Children* study officially began on June 1, 2016. Specific Aim 1 of the project was stated as “Finalize the adaptation of the Family Check-Up 4 Health (FCU4Health) program, which was initially adapted and piloted in pediatric primary healthcare, based on input from a CAB and partner clinics.” In brief, the trial is an effectiveness-implementation hybrid trial comparing an integrated/co-located model of care with a referral to external services model in partnership with three primary care agencies. Families are randomly assigned to FCU4Health (*n* = 200) or to clinic services as usual plus information (*n* = 150). The FCU4Health protocol in the *Raising Health Children* study consists of three family health behavior assessments and three feedback sessions, with individually-tailored family support sessions and referral to community-based services, over a 6-month period. An assessment 1 year after baseline will be used to examine lasting effects. The full study protocol is available in Smith et al. (26). As of this writing, the trial is still enrolling participants and providing the FCU4Health to families randomized to that arm. FCU4Health coordinators and supervisors meet regularly to discuss barriers and refinements to delivery process.

#### Community advisory board

The CORD 2.0 funding mechanism entailed the inclusion of a CAB with the goal of ensuring that at the conclusion of the project, the program would be ready for dissemination. The request for applications stated, “Collaboration with state CHIP and/or Medicaid offices to advise a state-wide or regional level project and to be part of a stakeholder group that can help generate suitable recommendations for sustainability and program components to be further replicated or scaled.” In addition to a representative from the state Medicaid office, known as the Arizona Health Care Cost Containment System (AHCCCS), our CAB includes leadership and direct service providers from local agencies (including our partner agencies for the project); stakeholders from relevant local entities (e.g., local health department, Arizona chapter of the American Academy of Pediatrics); representatives from health insurance plans; and researchers in obesity prevention, nutrition, and health disparities. We used community engaged dissemination and implementation research methods (65) to inform the conduct of our research and the execution of implementation.

We convened our first official CAB meeting in May 2016 (3 h) to prepare for the project starting. We next convened a day-long CAB meeting in September 2016, in which we held three concurrent work groups with the aim of obtaining guidance on three key aspects of the project: (1) evidence needed for post-project adoption; (2) integration of FCU4Health into the pediatric primary care system; and (3) program components to increase effectiveness for prevention of excess weight gain. Clearly, the latter two are directly relevant to adapting the program's delivery and enhancing its content. Berkel et al. (Berkel, C., Rudo-Stern, J., Villamar, J., Wilson, C., Flanagan, E., Smith, J.D. *Recommendations from community partners to promote sustainable implementation of evidence-based programs in primary care*, manuscript in preparation) discuss our partnership formation and the products of the CAB through the qualitative analysis of these work groups. Relevant findings for adaptation and enhancement are presented in the Results section. Recently, the CAB and four collaborators from the Obesity Prevention and Control Branch at the CDC, assembled for a 3 h meeting in September 2017 for updates on project progress and a discussion of successes and challenges to achieving the stated aims.

## Results

This section of the paper describes the FCU4Health program and classifies in what ways the original FCU was adapted for primary care or enhanced for the prevention of obesity and excess weight gain. Importantly, we also note important aspects of the FCU that were not changed for FCU4Health; these were critical for maintaining fidelity to the core components of the program and the underlying theory of change. We use the framework and coding system for modifications to EBPs developed by Stirman et al. (25). There is also a description of the activities (listed in the Method section) that were used in making the described adaptations. Finally, we use the JaKa et al. (27) taxonomy to specify the behavior change techniques used in FCU4Health as a means of providing a standardized comparison to similar programs.

### Adaptation and enhancement

The Stirman et al. (25) framework considers first the type of modification: *content* of the EBP or *context* in which it is being delivered. Within content modifications, 12 categories concerning the nature of the modification are identified and the level at which the modification occurs is specified (e.g., individual patient, clinic population). For context modifications, 5 categories were identified and include changes to the format, the setting, or the patient population (that do not result in changes to the actual content of the EBP). For each type, who was responsible for the modification is also indicated. Table 1 presents our classifications of the context modifications and Table 2 the context modifications made to the original FCU in development of the current model of FCU4Health. The narrative that follows in this section is intended to supplement and synthesize the information in the table by providing information on the timing of the modification and the sources of data that were used. This is one aspect of the Stirman et al. framework where we diverge. As it was intended to be applied retrospectively and not prospectively, we note when modifications were a priori (by the program developers) or after data sources indicated a need. The narrative also covers a third area of modification in the Stirman et al. framework: training and evaluation.

**Table 2:** Classifications of CONTENT modifications to the original FCU in developing the FCU4Health program.

**What is modified?**		**By whom were modifications made?**	**Level of delivery**	**Nature of the modification**
**FCU**	**FCU4Health**			
Screening—child behaviors and family risk factors for ineffective parenting	Screening—child body mass index (BMI)	Program developers Researchers (required by funding agency)	System level	Substituting
3 contacts (initial interview; ecological family assessment; feedback session)	2 contacts (combined initial interview and family health routines assessment; feedback session)	Program developers	System level	Shortening/condensing
Ecological assessment surveys—focused on ecological influences on children's adaptations and behavior and on parent's ability to manage the family	Family health routines surveys –added health routines and behaviors module (e.g., dietary practices, mealtime and sleep routines, physical/ sedentary activity, health related quality of life)	Program developers Researchers Coalition of stakeholders	System level	Adding elements
Family Interaction Tasks—focus on risk reduction of factors related to problem behaviors (e.g., monitoring, limit setting) 5 tasks, 5 min each	Family Interaction Tasks—focus on promoting healthy goals/behaviors and setting limits on unhealthy behaviors 3 tasks, 3-4 min each	Program developers Coalition of stakeholders	System level	Substituting Tailoring/tweaking/ refining
N/A	Anthropometric evaluation (BMI, body composition) of child and other family members, who are encouraged to provide this data	Program developers Researchers (required by funding agency) Coalition of stakeholders	System level	Adding elements
Referrals to community services and supports	Referrals to community services and supports Programs and services for diet, nutrition, physical activity and services to address social determinants of health	N/A Program developers Coalition of stakeholders	System level System and clinic/unit levels (tailored at the individual patient level)	N/A Tailoring/tweaking/ refining
No explicit focus on nutrition or health behaviors related to obesity and excess weight gain	Nutrition and child health behavior education and goals/expectations	Program developers Coalition of stakeholders	System level (tailored at the individual patient level)	Adding elements

#### Context modifications: adaptation for primary care

The 1-on-1 delivery format of the FCU and use of home visitation during early childhood was retained for FCU4Health. The motivation for and merits of a 1-on-1 approach that occurs largely in the family home, compared to the common group-based delivery of parenting programs at a central location, are discussed in Smith et al. (53) and Dishion and Kavanagha (48). Home visiting for delivery of behavioral health is particularly germane to coordinating with pediatric primary care due to the space limitations of typical medical offices for use by behavioral health staff. In FCU4Health, identification, referral, and initial contact (ideally) occur in the primary care office and the remaining intervention services predominantly occur in the family home or a community location (e.g., community center, YMCA). However, the delivery strategy is flexible and is currently being done in multiple ways in an ongoing trial aligning with the staffing, space, and preference of the clinics involved (Berkel et al., submitted). One major format modification that was made for the Raising Healthy Children study concerns the intensity of services provided. The original FCU was designed for selected and indicated prevention and was intended to be delivered using a health maintenance approach. Specifically, each year the family has a comprehensive assessment and a “feedback session” to build motivation and plan follow-up services for which the intervention intensity (number and frequency of sessions) is guided by the current level of need (66). In this project, a more intensive model of delivery is being used. The CORD 2.0 RFA required a delivery approach that would meet the recommended 25 to 50 h of intervention time over a 6-month period specified by the US Preventive Services Task Force for youth with a BMI for age and gender of ≥85th percentile (67). This requirement led us to devise a condensed health maintenance approach with three feedbacks in 6 months (Months 1, 3, 6), rather than the customary annual feedback, to facilitate achieving the hourly target, allow us to continually tailor the intervention for each family, and explicitly address motivation to change behavior—a primary challenge in family-based intervention for the prevention of excess weight gain (68) and an explicit target of the FCU and FCU4Health. This schedule aligns with the suggested frequency of visits to primary care for children with obesity (69). In this way, the FCU4Health is being delivered as an indicated intervention in the Raising Healthy Children study. However, in an ongoing trial of FCU4Health funded by the United States Department of Agriculture to Berkel and Smith (Grant number 016–10799), it is being delivered as a selected intervention for young children (ages 2 to 8 years) who screen positive for poor dietary habits but who do not have an elevated BMI. Rather than the intensive delivery of the program as is being done in the Raising Healthy Children study, delivery of FCU4Health occurs annually for 3 consecutive years with individually-tailored intervention plans (i.e., number of hours each year vary from 3 to 10) to correspond with each child and family's specific level of need.

Concerning the setting, we have previously discussed our scale-out effort to primary care. The program developers sought to take the FCU into this service context for a number of reasons. First, it is a setting that serves a high proportion of children and families; parents are typically present at children's healthcare visits; and parents are used to receiving advice from pediatricians as a trusted source of information (7). These factors generally support a parenting intervention in primary care. Specific to shifting our clinical target of obesity and excess weight gain, primary care is a context where weight and weight-related behaviors are thoroughly embedded, it is the only system that regularly tracks weight throughout childhood, and parents may be more receptive to learning about their child's risk for obesity from their pediatrician than in other contexts (7) where identifying children with elevated BMI creates concerns about confidentiality and stigma (70). Our early and ongoing meetings with stakeholders, survey of pediatricians' needs, and pilot trial provided the necessary evidence that such a program is acceptable and appropriate for this setting. Formal data collection on acceptability and appropriateness is ongoing in the *Raising Healthy Children* study, but no major concerns have emerged up to this point.

The personnel that typically deliver FCU were largely maintained for FCU4Health. The primary providers are Master's-level clinicians with backgrounds in mental and behavioral health. In working with our partner agencies, and discussing children's primary healthcare practices more broadly with the CAB, we elected to allow professionals from obesity-related fields, such as health promotion, nutrition, and public health, to be trained to deliver the intervention, as these are the professional roles that serve similar functions to FCU4Health in pediatric healthcare agencies and often have training in motivational skills. Further, some components of the FCU4Health (i.e., conducting the assessment, connecting families with referrals to community resources to address contextual needs) may be completed by community health workers or promotoras. This diffusion of responsibilities fits with the medical home framework in which each person in the clinic performs roles in accordance with their training and abilities (71). The procedures for implementation vary, however, by the agency or clinic depending on their available personnel and other resources and the model of behavioral health services used (e.g., integrated care, coordinated care, colocation, referral to external service provider). Thus, when implementing FCU4Health, there is a need to accomplish specific program activities but the manner in which this is done, who is responsible, and even where they are delivered—in the clinic, the home, or another agency's offices—is flexible (bib70; Berkel et al., submitted).

With the change of setting came a change in the referring professional. In previous trials, referrals originated with school personnel, the parents, or a mental health provider. In keeping with typical procedures in pediatric primary care, which was also a requirement of the CORD RFA, the pediatrician identifies children with elevated BMI and refers to the FCU4Health. In our pilot trial and in meetings with our partner clinics and the CAB, this procedure was found to be feasible and appropriate.

Population modifications centered on the new clinical target: pediatric obesity. Instead of the FCU procedure of targeting characteristics of children and families focused on risk reduction for problem behaviors, FCU4Health targets families with youth at risk for obesity and excess weight gain, but with a health promotion approach. Characteristics considered include behavioral risk factors, such as poor dietary practices and low physical activity, and also membership in sociodemographic groups that are disproportionately affected by the obesity epidemic, including low-income and racial/ethnic minority families (73). The age of the targeted youth is intended to be the same as the original FCU, which is 2 to 17 years, however, CORD 2.0 funding is for inclusion of children ages 6 to 12 years.

In summary, nearly all context modifications were made by the program developers, who are also the researchers on the project. Some modifications were directly influenced by the CORD 2.0 RFA. Agency administrators (i.e., leadership at our partner clinics) were influential in the decision to include related professionals in the delivery of FCU4Health. Nearly all of these modifications were determined a priori to the grant proposal, based on our prior experiences (e.g., meetings, pilot trial).

#### Content modifications: enhancement for pediatric obesity

Content modifications to FCU primarily involved tailoring and adding elements to address obesity and health behaviors. Care was taken to retain core components of the FCU in order to maintain its effectiveness at improving parenting and family functioning, which we found mediated effects of the program on obesity in childhood and adolescence to adulthood (41, 43). Content modifications were made in close collaboration with our CAB. Berkel et al. (manuscript in preparation) report the primary qualitative results of an analysis of transcripts from three working groups conducted at our September 2016 CAB meeting. These working groups discussed the topics of (1) fit of the FCU4Health within primary care, (2) components of the program for prevention of obesity and excess weight gain and management of co-occurring concerns, and (3) evidence needed to support sustainment of the program after the trial. Salient results from this qualitative research are included in the following sections.

The procedures for delivering FCU4Health differs somewhat from FCU due to the demands of the delivery setting and the new clinical target. Members of our CAB engaged in a working group on the issue of fitting FCU4Health into primary care. The primary themes concerned fit with the clinic's mission and needs, clinic staffing, and patient characteristics. To this end, we first needed a new screening process with the shift to prevention of obesity and excess weight gain necessitated. Although the CORD RFA dictated that the pediatrician was to use the child's BMI to initiate referral to FCU4Health after providing counseling, consistent with Healthcare Effectiveness Data and Information Set (HEDIS) procedures for children with BMI ≥ 85th percentile for age and gender, our pilot trial experience and CAB work group on integration confirmed that these steps and personnel aligned with clinic practices and were preferred. This modification was a substitute for the FCU screening for child problem behaviors (e.g., oppositionality) and family risks for ineffective parenting (e.g., parental depression). Next, we shortened/condensed the number of contacts between the family and the FCU4Health coordinator for the “check-up” portion of the program based on pilot data indicating that families had difficulty completing the typical 3 sessions of the FCU and preferred fewer contacts (63). FCU4Health combines the initial interview and assessment^2^, whereas these were originally separate meetings in FCU.

Modifications to the family assessment, which were mostly additions, were fairly extensive. In the original FCU, an ecological family assessment is conducted to gather information on the various influences on both child problem behaviors and on parenting effectiveness (24). The majority of the constructs and items in the original assessment were retained because they are relevant to health behaviors (e.g., child self-regulation) or to parenting (e.g., social support, parental depressive symptoms). However, a health module was added to the survey portion of the assessment to gather more pertinent information about the constructs of (a) child dietary habits; (b) family health routines (mealtimes, sleep, media) and behaviors (dietary practices, exercise); (c) health-related quality of life; (d) weight-related stigma; (e) body image; and (f) the management of common co-occurring health conditions when present (i.e., asthma, diabetes). These additional constructs were in part a result of a working group meeting of our CAB on components of the FCU4Health. In this meeting weight-related stigma came up as an area of particular importance, as did the need for referral resources to support child and family health in the areas listed previously.

The FCU assessment also includes an observational component to rate parenting skills and family functioning using the Family Interaction Task (FIT), which is a series of semi-structured family interactions that are coded using a validated system (74). In the spirit of shortening the FCU4Health, we modified the number, length, and prompts of the FIT. In FCU, five tasks (5 min each) were administered with a focus on factors related to preventing child problem behaviors (e.g., monitoring the child's whereabouts and peer network). In FCU4Health, we administer three tasks (4 min each) concerning health goals and promoting healthy behaviors. For example, the instructions for the Goal Setting task are:

To Child: I'd like you to talk about your goals for yourself for exercise and your diet, especially developing healthy habits. Then, please talk about how you feel that it is going right now.

To Caregiver(s): When (child name) is finished, please talk about your goals for his/her health, diet, and exercise behavior. Share with (child name) some specific ways you plan to help support those goals. Then please talk about your hopes and plans for your son's/daughter's future health.

The same coding system is used to assess parenting skills with one salient addition: parents' knowledge of national guidelines for children's health behaviors. Examples include the current recommended amount of daily physical activity, servings of fruits and vegetables, and amount of screen time. The FCU4Health developers piloted a version of these FIT prompts in the pilot trial and they were refined with guidance from our partner agency staff and the CAB.

A final addition to the family health routines assessment for FCU4Health was anthropometric evaluation of the child and the family members using a portable medical-grade electronic scale to obtain weight and body composition data. In the grant application, we proposed to capture these data from the child only. There was a concern with respect to our economic assessment as to whether we would see cost-benefit within 1 year. Because adult BMI is more proximally linked with expensive health outcomes, and because we theorized that by promoting healthy diet and physical activity in the family, parents may also experience reductions in obesity. The CAB felt that having the entire family get on the scale would normalize the measurement process for the child. Consequently, we decided to add parent weight and body composition to the assessment and encourage being weighed and measured. As weight was not a target of the original FCU, this element was simply added^3^.

The purpose of the assessment is to identify services that would help families support child health and motivate parents to engage in those services. These follow-up services can take one of two forms. To address needs related specifically to parenting, the coordinator provides parenting skills training using *Every day Parenting* (75), a 12-module skills-based curriculum focusing on three core areas of parenting and family management: relationship quality, positive behavior support, and monitoring and limit setting. This element was refined from the FCU, which also shares this explicit goal, by using examples that specifically focus on health behaviors (e.g., setting limits on screen time). In both programs, the number of *Every day Parenting* modules and the type and number of referrals for community-based support services are individualized to the specific needs of each family following a feedback session that discusses the key findings of the family assessment. Although FCU4Health adheres to the *Everyday Parenting* modules as they pertain to skill-building, because of the program's target, coordinators add a focus on children's nutrition and age-appropriate health behavior expectations. This element aligns with our added category in the FIT assessment of parent understanding of health guidelines. FCU does not provide this information as part of standard protocol.

To address other areas of need, the coordinator shares information about resources in the community and provides motivational and logistical support to families to connect with those resources. In FCU4Health, there is an emphasis on referrals to health-related community supports, such as food banks, community gardens, and recreational programs, and also to social services that can help the family address social determinants of health related to childhood obesity (76). There is an explicit goal of assisting families in procuring insurance for their child(ren) and securing or maintaining employment. Similar to the original FCU, FCU4Health also commonly refers parents to specialty mental health services, when indicated, for such issues as children's mental health concerns (e.g., developmental delays, attention deficit hyperactivity disorder) parental depression and substance use. FCU4Health has a greater focus on specialty health care for common co-occurring conditions, most notably chronic health conditions such as asthma and diabetes. The CAB workgroup meeting on program components yielded recommendations on how to compile, maintain, and disseminate up-to-date information on referral resources to facilitate referrals.

#### Training and evaluation modifications

The FCU4Health training and supervision process and implementation monitoring system remains largely consistent with the most recent trials of FCU. Three aspects differ from prior trials of FCU. First, given that the *Raising Healthy Children* project is an effectiveness trial, the amount of consultation from FCU4Health developers and supervisors, and ongoing oversight more generally, is less prescribed in amount and duration compared to efficacy trials by Dishion and colleagues [e.g., Dishion et al. (24)]. The best comparison to our procedure and amount of training and consultation is an effectiveness-implementation hybrid type I trial conducted with the original FCU in community mental health agencies (54). A second modification to training compared to previously published research trials is the use of an e-learning course developed by Dishion and colleagues at the ASU REACH Institute as the prerequisite to in-person training in the program. The e-learning course is on the original FCU (77) and the *Everyday Parenting Curriculum* (78) and covers the theoretical background and core components of the parenting aspects of the program; we focused on supplementing this information with the health-related adaptations of the FCU4Health during the in-person training. Third, FCU uses a validated, observational coding system called the COACH (79) to monitor delivery of the program. The COACH is an observational rating system of fidelity in delivering FCU4Health. Skills in five areas (Conceptually accurate to FCU4Health; Observant and responsive to client needs; Actively structures the session; Corrective feedback is provided; Hope and motivation) are rated on a scale from 1 (low) to 9 (high) by trained coders. Scores on the COACH have been found to be reliable and related to change in both parenting skills and child behaviors in previous trials (80–82). For *Raising Healthy Children*, we are using the COACH, but are also developing an automated system to rate fidelity based on existing, validated systems for core elements of motivational interviewing (e.g., presence of complex reflections, open-ended questions) (83–85) and other family-based and parent training interventions (bib84, Li et al., submitted). This system will allow us to evaluate fidelity to FCU4Health for every session rather than the typical practice of coding a small sample due to the burden of observational assessment. Thus, the training of FCU4Health includes: for coordinators, completion of a 7-module e-learning course, a 3-day in-person training, completion of a mock case, and close supervision for the first two families seen is encouraged but not required, and varies based on ratings of fidelity to the protocol; for interviewers (those completing the assessments), a 1-day training that includes a practice administration; and for referring physicians and other healthcare staff and leadership/managers in the clinics, a 30–45 min orientation to the program and the referral procedures and inclusion criteria.

#### Specifying the behavior change techniques of the fcu4health

**Figure 3:**
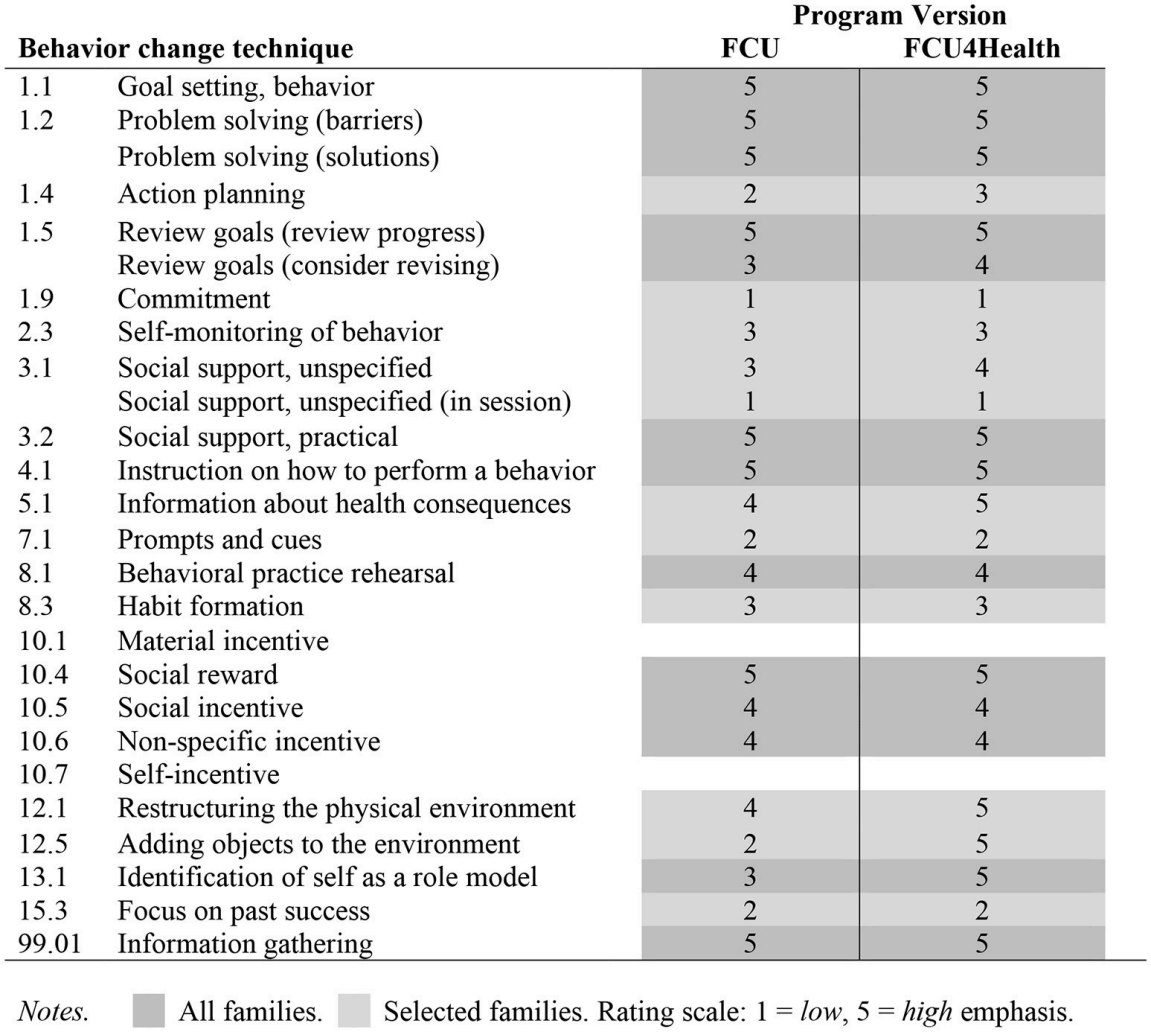
Specification of behavior change techniques in FCU4Health and the original FCU. All families. Selected families. Rating scale: 1, low; 5, high emphasis.

JaKa et al. (27) developed a standardized protocol to specify the type and amount of behavior change techniques used in behavioral interventions for pediatric obesity. They drew from the original 93 techniques in the Behavior change Taxonomy (87). For the purposes of this article, we specify the type and rate the emphasis given each technique on a scale of 1 (*low*) to 5 (*high*). JaKa et al. also code the amount of each technique, but this can only be determined from observation of the program's delivery. Figure 3 provides a side-by-side comparison of the FCU4Health and the original FCU. Because the FCU4Health is individually tailored to the needs of each child and family, we further specify whether a given technique is universal (received by all families in the program) or applied selectively based on needs identified in the family health behaviors assessment. We present the 23 techniques Jaka et al. (27) found to be reported at least once in their evaluation of intervention protocols, manuscripts, and workbooks, indicating salience for childhood obesity programs whereas the remaining 70 techniques are unlikely to be relevant. Further, given that FCU4Health is a family-based intervention, certain behavior change techniques are taught to caregivers to then use with the child. For example, we train caregivers to provide effective social rewards to the child to reinforce desired behaviors.

## Discussion

This paper presents the adaptation of an evidence-based prevention program to scale-out to a new delivery context and for a new clinical target. The well-established FCU program was adapted for the pediatric primary care context and enhanced to more effectively prevent obesity and excess weight gain in children. The resulting program, FCU4Health, is likely to be acceptable and feasible based on pilot study data (63) and is currently being tested in a large randomized effectiveness-implementation hybrid trial (26) to gather this data alongside evidence of clinical impact on children's weight and health behaviors.

A number of key changes were made when developing FCU4Health, while other core components and characteristics of FCU were retained. C*ontext adaptations*, most of which were made a priori by the program developers, included the identification and referral process; a shift to a health promotion focus; reducing the number of total contacts for the “check-up” component; and a division of responsibilities among clinic staff for the various components of the program. Important context characteristics that remain unchanged from the original FCU are an emphasis on underserved children disproportionately at risk for the target outcome; the coordinator's being behavioral health professionals; 1-on-1 delivery format to maximize flexibility of delivery and reduction of barriers to maximize participation; and engaging caregivers in multiple “check-ups” to track progress and continually enhance motivation to change behaviors. *Content adaptations* were almost exclusively due to the change in clinical target. These additions began during pilot testing of the FCU4Health and were later refined in collaboration with the CAB. A key modification was making the family assessment more relevant to weight-related behaviors. Importantly, the critical processes that underlie change in the FCU were retained. These include assessment, feedback, motivation enhancement, coordination with community supports and programs, and individualized intervention planning. Training, supervision, and implementation monitoring largely remains unchanged, with the exception of using a recently-completed e-learning course to train coordinators and developing an automated fidelity coding system as part of the ongoing *Raising Healthy Children* study.

Adaptation is a commonly used implementation strategy to better align EBPs with the characteristics of real-world service delivery systems and the populations being served (18). Despite the prevailing use of EBP adaptation in practice, this paper provides a number of unique contributions to the implementation research literature, such as how adaptations are characterized, for what purposes, and how the decisions to adapt were made. First, we framed our adaptation process and aims on the new implementation science concept of scaling out (15). In contrast to the common approach of incremental adaptation typical in the existing literature, where modification to either the delivery context or to the population is described, this paper is an example of simultaneous adaptation to both dimensions, which speeds translation of EBPs. We also delved deeper into the scaling out concept in an important way by providing detailed, hierarchical levels of evidence to be applied when scaling out involves adapting a program for a new clinical target. By providing Minimal, Preferred, Preferred Plus, and Optimal levels of evidence, researchers, reviewers, and stakeholders can better evaluate the case for scaling out in this manner. In combination with available support for changing delivery context or population (e.g., age, racial/ethnic group), the level of evidence can be used to justify scaling out to a new clinical target. Future work could involve similarly specifying levels of evidence for changing to a new delivery context or population characteristic (without changing the clinical target). Currently, this does not exist explicitly.

Second, we used the Stirman et al. (25) adaptation coding system in a novel way to characterize the types of adaptations made, by whom, and based on what sources of data. Stirman et al.'s system provides a framework for a comprehensive description of adaptations. We found it to be particularly useful for the current paper as it was intended to be applied retrospectively. In contrast to the typical use of the system for coding individual sessions, we were able to successfully apply it to the program as a whole using a common language that other adapters could also use to describe their adapted EBPs. The consistent use of terminology is a critical challenge in implementation research (88).

Third, we provide a comparison of the behavior change techniques used and their levels of emphasis and application between the original FCU and the new FCU4Health program. We used the techniques that JaKa et al. (27) identified as most common in EBP protocols of behavioral interventions for pediatric obesity. Specification of techniques in this manner helps to open the “black box” of how these interventions work and allows for comparison with the active ingredients of other, similar programs. In this paper, it was also useful in highlighting the similarities and the differences between FCU and FCU4Health. The differences were minor and centered on a greater emphasis in FCU4Health on changing the physical environment and the caregiver being a role model for healthier child behaviors. These minor differences provide support for our assertion that the core components responsible for the effectiveness of the original FCU were retained in FCU4Health. Last, without the ability to rate how frequently each technique was used in FCU4Health delivery, as the JaKa et al. rating system was intended, we used an emphasis scale and indicators of either to all families or to select families to further illustrate the degree of likely use. Observational coding of FCU4Health sessions in the future could be done to quantify with better precision the frequency at which each technique is used.

## Considerations

One of the challenges in both adapting the FCU and in describing it in this paper is that the program is individually-tailored and delivery is flexible by design. This made it challenging to code adaptations; many of the elements of FCU4Health would be acceptable if done within the context of the original FCU. For example, discussing a need for more physical activity and less screen time in FCU would be appropriate if it related to a concern raised by the caregivers even if it's not an explicit target of that program. FCU4Health more or less uses the core intervention techniques and process of the FCU, but shifts the focus to a new clinical target, pediatric obesity, and emphasizes parental management and supports to improve child health behaviors. While many adaptations described here could be considered fidelity-congruent within FCU, but not necessarily prescribed, they should be considered necessary for high fidelity to FCU4Health given the new clinical focus.

From a practical perspective, the elements that we added to FCU4Health's questionnaires increased the time to complete, particularly for children with additional chronic health conditions (the presence of asthma and diabetes trigger additional questions). The family assessment is already a challenge to complete in many service delivery systems due to the time required. Thus, we expect in the future to pare down the assessment to its necessary constructs based on the findings of this study to reduce burden on families and agencies. This consideration harkens back to the framework of scaling out to a new delivery context and the need to consider capacity and readiness to adopt and deliver an EBP. Although assessment is commonplace in pediatric primary care, the measures are typically screeners that are very short. Moreover, assessments are sometimes administered via semi-structured interview format where pediatricians write out responses. Thus, it might be challenging to change this practice in favor of the FCU4Health questionnaire and FIT assessment even if the time required is comparable.

## Conclusions

This paper provides a detailed account of the many sources of information and data that inform an ongoing process of adaptation to meet the changing needs of the setting and the population served. Our approach aligns well with the Dynamic Sustainability Framework (13) and is an example of community engaged dissemination and implementation (65). Our process to date occurred over 6 years and will continue. Adaptations have and will continue to occur as we triangulate data from multiple sources (e.g., delivery, feedback from stakeholders, examination of clinical effects). As the FCU4Health is implemented, we are continuing to refine the program components and delivery strategies with input from our CAB, the partner agencies, FCU4Health coordinators, and our implementation support staff. A key activity as the *Raising Healthy Children* study nears completion is to review our implementation data and work with stakeholders, including caregivers and children, to determine what the program will look like and how it will be delivered in the “real world”; that is, as the agencies attempt to sustain implementation of FCU4Health outside of a formal research study. We expect the process of adaptation to be ongoing as the healthcare landscape for children evolves and the priorities of the agencies that serve them also shift.

## Author contributions

JS and CB conceived of the study. JS, CB, JR-S, ZM, AM, AC, MB, and TD participated in different phases of the adaptation process and contributed to the adaptation of the FCU in substantive ways. JS, CB, and JR-S collaborated in the writing of the manuscript and wrote the final manuscript. All authors have read and approved the manuscript.

### Conflict of interest statement

JS and CB led the adaptation of and co-developed the Family Check-Up 4 Health program along with TD. TD is the developer of the original Family Check-Up program. The remaining authors declare that the research was conducted in the absence of any commercial or financial relationships that could be construed as a potential conflict of interest.

## Footnotes

^1^The FCU4Health as described in this article is being tested in the *Raising Healthy Children* study compared to primary care services as usual.

^2^In the *Raising Healthy Children* study, we conduct the assessment first in a stand-alone contact in order to double-blind the collection of baseline data prior to randomization and follow-up assessments at later waves. The initial interview is then combined with the feedback. In routine implementation of FCU4Health, the initial interview and assessment would be combined.

^3^In a previous trial of FCU, children's height and weight data was collected as part of the research protocol, but this information was not used in the intervention in any way [see Montaño et al. (42)]. Thus, we consider its explicit use in the FCU4Health an addition.

## Abbreviations

ASU: Arizona State University
CAB: community advisory board
CDC: Centers for Disease Control and Prevention
CHIP: Children's Health Insurance Program
CORD: Childhood Obesity Research Demonstration project
EBPs: Evidence-based programs and practices
FCU: Family Check-Up
FIT: Family Interaction Task
FCU4Health: Family Check-Up 4 Health
PCH: Phoenix Children's Hospital.
